# Ipl1/Aurora Kinase Suppresses S-CDK-Driven Spindle Formation during Prophase I to Ensure Chromosome Integrity during Meiosis

**DOI:** 10.1371/journal.pone.0083982

**Published:** 2013-12-27

**Authors:** Louise Newnham, Philip W. Jordan, Jesus A. Carballo, Sonya Newcombe, Eva Hoffmann

**Affiliations:** MRC Genome Damage and Stability Centre, University of Sussex, Brighton, United Kingdom; Oklahoma Medical Research Foundation, United States of America

## Abstract

Cells coordinate spindle formation with DNA repair and morphological modifications to chromosomes prior to their segregation to prevent cell division with damaged chromosomes. Here we uncover a novel and unexpected role for Aurora kinase in preventing the formation of spindles by Clb5-CDK (S-CDK) during meiotic prophase I and when the DDR is active in budding yeast. This is critical since S-CDK is essential for replication during premeiotic S-phase as well as double-strand break induction that facilitates meiotic recombination and, ultimately, chromosome segregation. Furthermore, we find that depletion of Cdc5 polo kinase activity delays spindle formation in DDR-arrested cells and that ectopic expression of Cdc5 in prophase I enhances spindle formation, when Ipl1 is depleted. Our findings establish a new paradigm for Aurora kinase function in both negative and positive regulation of spindle dynamics.

## Introduction

The DNA damage response (DDR) prolongs the G2/M or prophase arrest when cells are challenged with DNA damage. This is important to prevent attempts at chromosome segregation in the presence of DNA damage that would compromise the genomic integrity of cells. In meiosis, the importance of DNA repair and cell cycle progression has recently been demonstrated in human oocytes, where decreased capacity for DNA repair correlates with reduced ovarian reserve [1]. Even without DNA damage, there are several examples where prophase I is extended, most notably the decades-long prophase I/dictyate arrest in human oocytes. In budding yeast, meiotic prophase I is extended ∼ 10-fold compared to mitotic cell cycle [2]. This allows the induction of 150–200 double-strand breaks (DSBs), whose repair by homologous recombination facilitate efficient homolog pairing and crossing over prior to the two nuclear divisions [3], [4]. Modifications to chromosome morphology and behaviour are also required to set up the two consecutive segregations of first homologous chromosomes (meiosis I), followed by sister chromatids (meiosis II).

In budding yeast, a single cyclin-dependent kinase (CDK/Cdc28) drives the cell cycle together with six B-type cyclins (Clb1-6). Clb5,6-Cdc28 (S-CDK) promotes DNA replication and spindle pole body maturation (the yeast microtubule organizing centers), whereas mitotic and meiotic divisions are promoted by Clb1,2,3,4-CDK (M-CDK) [5], [6], [7]. Clb2 is tightly repressed throughout meiosis [8], [9]. After meiotic entry, Clb5 and Clb6 are present at low levels throughout meiosis and Clb5 is required for DNA synthesis as well as DSB induction by Spo11 [10], [11], [12]. Clb5 mutants display low sporulation efficiency, whereas Clb6 has no detectable defects [10]. This is consistent with the notion that Clb5 is the main facilitator of S-CDK activity during meiotic prophase I.

**Figure 1:**
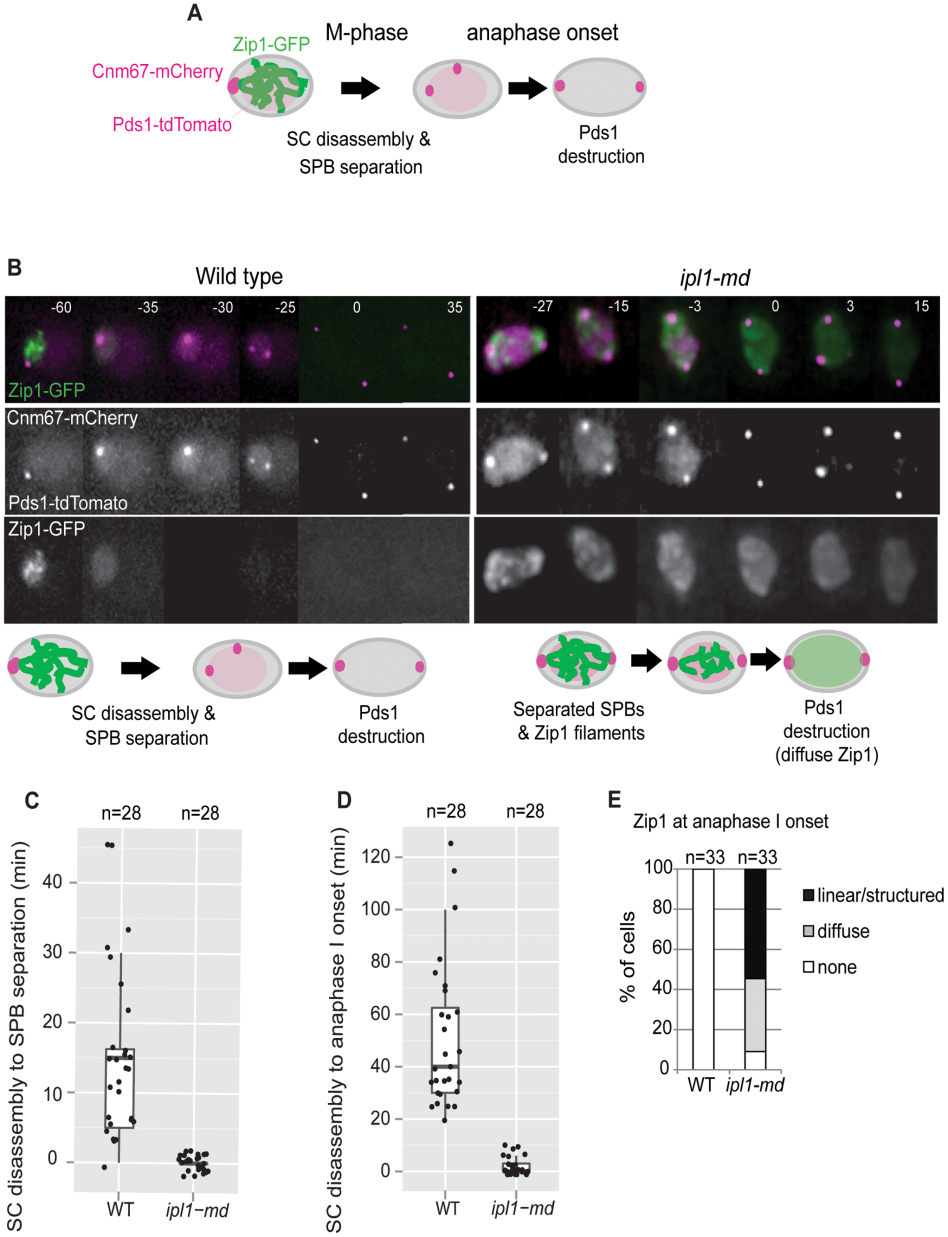
Zip1 disappearance is delayed in *ipl1-mn* mutants. (A) Experimental set up. SCs are followed by Zip1-GFP; SPBs by Cnm67-mCherry, and anaphase I onset by Pds1-tdTomato (confluent staining). (B) Representative montages of SC disassembly (loss of Zip1-GFP), SPB separation (CNM67-mCherry), and anaphase I onset (degradation of Pds1-tdTomato) in wild type (Movie S1) and *ipl1-mn* (Movie S2). Bars: 2 µm. (C) The proportion of cells with linear Zip1-GFP structures, diffuse Zip1-GFP staining or no Zip1-GFP signal at anaphase I onset (Pds1 degradation). (D) Time from SC disassembly (Zip1-GFP signal loss) to separation of the SPBs. (E) Time from SC disassembly to anaphase I onset (Pds1 degradation). Strains: WT, Y4044 and *ipl1-mn*, Y4047.

Onset of M-phase is regulated by the meiosis-specific Ndt80 transcription factor that induces expression of the M-phase cyclins, Clb1 and Clb4 [9], [10], [13]. Ndt80 is negatively regulated by the meiotic DDR and when active, drives cells from mid-prophase I (pachytene) into the meiotic divisions [14], [15]. Ndt80 is essential for extending prophase I and coupling prophase I exit to the DDR. Its mitotic counterpart, Ndd1, is actively degraded during meiosis and its stabilization causes a contraction in prophase and precocious expression of M-CDK and polo kinase, leading to meiotic catastrophe [16] (Fig. 1A). High levels of expression of Clb1, Clb3, or Clb4 can drive spindle formation, even when ectopically expressed in meiotic prophase I [17], [18]. This is consistent with the requirement for active CDK in SPB separation and spindle formation [19], [20]. In contrast, ectopic expression of Cdc5 polo kinase, which is up-regulated by Ndt80, leads to chromosome restructuring, but not SPB separation [20], although Cdc5 polo kinase activity is important for the timely separation of SPBs [21]. Thus, Cdc5 promotes the efficiency of, but is not sufficient to drive spindle formation. Combined, high levels of Cdc5 and M-CDK activity are thought to be required for spindle formation upon exit from pachytene.

Although the transcriptional activation of M-CDK is the main driver of spindle formation, S-CDK is active during all of meiotic prophase I [13]. In mitotically-dividing cells, S-CDK can drive spindle formation, albeit less efficiently than M-CDK [22]. This raises the intriguing question of how cells prevent S-CDK from promoting spindle formation during prolonged prophase I arrest in meiotic cells. Indeed, it has been reported that in *ndt80*Δ-arrested cells, Ipl1 depletion leads to spindle formation, including multipolar spindles [23], [24]. Here, we show that in cells in which Ipl1 is inhibited or depleted, S-CDK is both sufficient and necessary to promote spindle formation during meiotic prophase I, whereas Cdc5 Polo kinase assists in the efficiency of spindle formation. We infer that Ipl1 prevents precocious spindle formation by S-CDK and Cdc5. Consistent with the notion that precocious spindle formation is detrimental to establishing appropriate chromosome structure, the spindles that are formed in the absence of Ipl1 are highly dynamic and capable of triggering chromosome segregation and nuclear deformation [25].

## Results and Discussion

### Ipl1 Decouples Chromosome Restructuring and Bipolar Spindle Formation in Part by Preventing Spindle Formation during Meiotic Prophase I

In budding yeast, spindle formation normally occurs after disassembly of the synaptonemal complex (SCs), which is characteristic of pachytene/mid-prophase I. We previously demonstrated that cells depleted for the Aurora kinase orthologue, Ipl1 (*ipl1-meiotic depletion*), contained spindles in cells that displayed full SCs. Synaptonemal complexes (SCs) normally disassemble upon Ndt80-mediated exit from pachytene and entry into M-phase. However, in the Ipl1-depleted cells, the SCs were retained at later time points, despite the M-phase cyclins (Clb1 and Clb3) being expressed with wild-type timing [26], [27]. This led us to suggest that Ipl1 couples SC disassembly to cell cycle progression [27]. Recent observations suggest, however, that inactivation of Ipl1 causes a contraction in metaphase I [28], consistent with an earlier timing of the appearance of spindles in Ipl1-depleted cells. This, together with the observation that cells depleted for Ipl1 show precocious spindle formation, when held in *ndt80*Δ prophase I arrest [23], raises the distinct possibility that Ipl1 could also suppress precocious spindle formation in pachytene cells.

**Figure 2:**
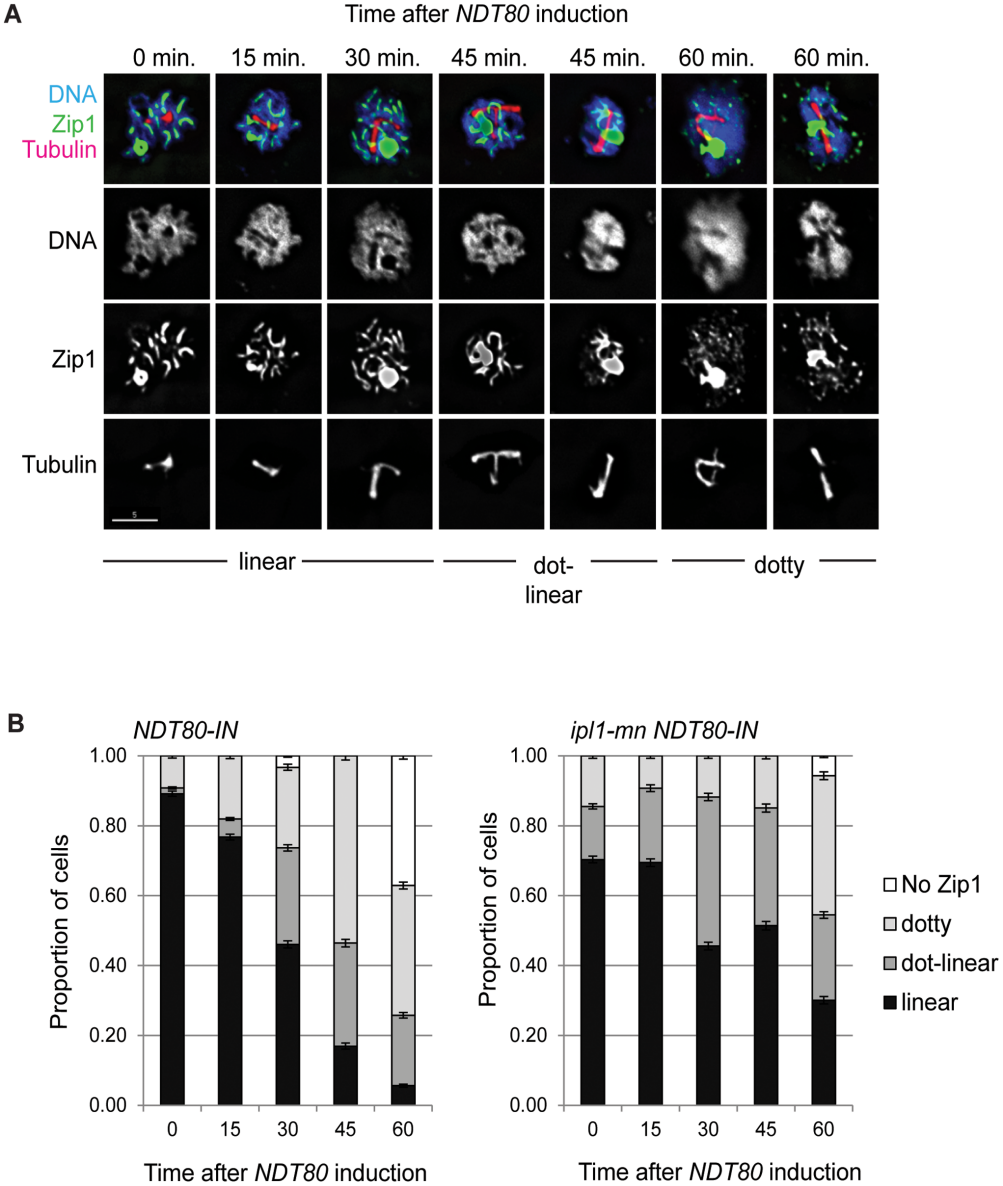
SC disassembly is delayed in *ipl1-mn* after release from Ndt80/mid-prophase arrest. (A) Examples of SCs, their classification and typical Zip1 staining patterns in the *ipl1-mn NDT80-IN* mutant during arrest (t = 0 min.) and after release from *ndt80* arrest. (B) Proportion of spread, meiotic nuclei with linear, dot-linear, dotty, or no Zip1 staining in *NDT80-IN* and *ipl1-mn NDT80-IN.* n >100 cells were assessed for each time point.

To investigate whether chromosome restructuring was delayed and/or spindle formation premature, when Ipl1 was depleted, we took advantage of developments in time-lapse imaging of the synaptonemal complex protein, Zip1-GFP [29], whose disassembly from the SC and degradation occur concurrently [27]. Spindle poles bodies (SPB) were marked by CNM67-mCherry, and anaphase I onset was monitored by Pds1-tdTomato degradation (Fig. 1, Movie S1). In the wild type, Zip1-GFP disappeared 15 min. (median time; n = 28) prior to SPB separation and 40 min. (median time; n = 28) prior to anaphase I onset (loss of securin/Pds1 signal, Fig. 1C). In contrast, virtually all of the *ipl1-md* cells contained a strong Zip1-GFP signal at the time of SPB separation as well as anaphase I onset (Fig. 1C–D, Movie S2). By anaphase I onset (Pds1 degradation), more than half of the cells still contained significant Zip1-GFP staining (Fig. 1E), including linear structures (Fig. 1E). We assessed fixed, spread meiotic nuclei as well to ascertain that the SCs observed were indeed associated with meiotic chromosomes (Fig. 2). Using fixed cells, we observed a delayed removal of SCs from the meiotic chromosomes after release from pachytene arrest (Fig. 2), as previously reported [27]. Collectively, our observations are consistent with those made previously in fixed, spread nuclei [27], and suggest that Ipl1 promotes coupling of chromosome restructuring with cell cycle progression. Since SCs eventually disassemble in metaphase-arrested Ipl1 mutants, Ipl1 promotes the efficiency [27], as opposed to being absolutely required, for chromosome restructuring. It is possible that the chromosome restructuring defects could be due to the contraction in the cell cycle *per se*, since metaphase I is shortened in *ipl1* mutants [24]. This would imply that cell cycle progression into M-phase of meiosis I occurs in parallel with SC disassembly and that cells have a limited window for chromosome restructuring. Moreover, the nature of cell cycle contraction clearly matters, since SC disassembly is not delayed relative to spindle formation in *mad3* mutants [27], where the meiotic cell cycle is also contracted [30].

### ipl1-md Mutants Display Spindle Formation in ndt80 and Efficient Spindle Formation after Entry into Meiosis I

**Figure 3:**
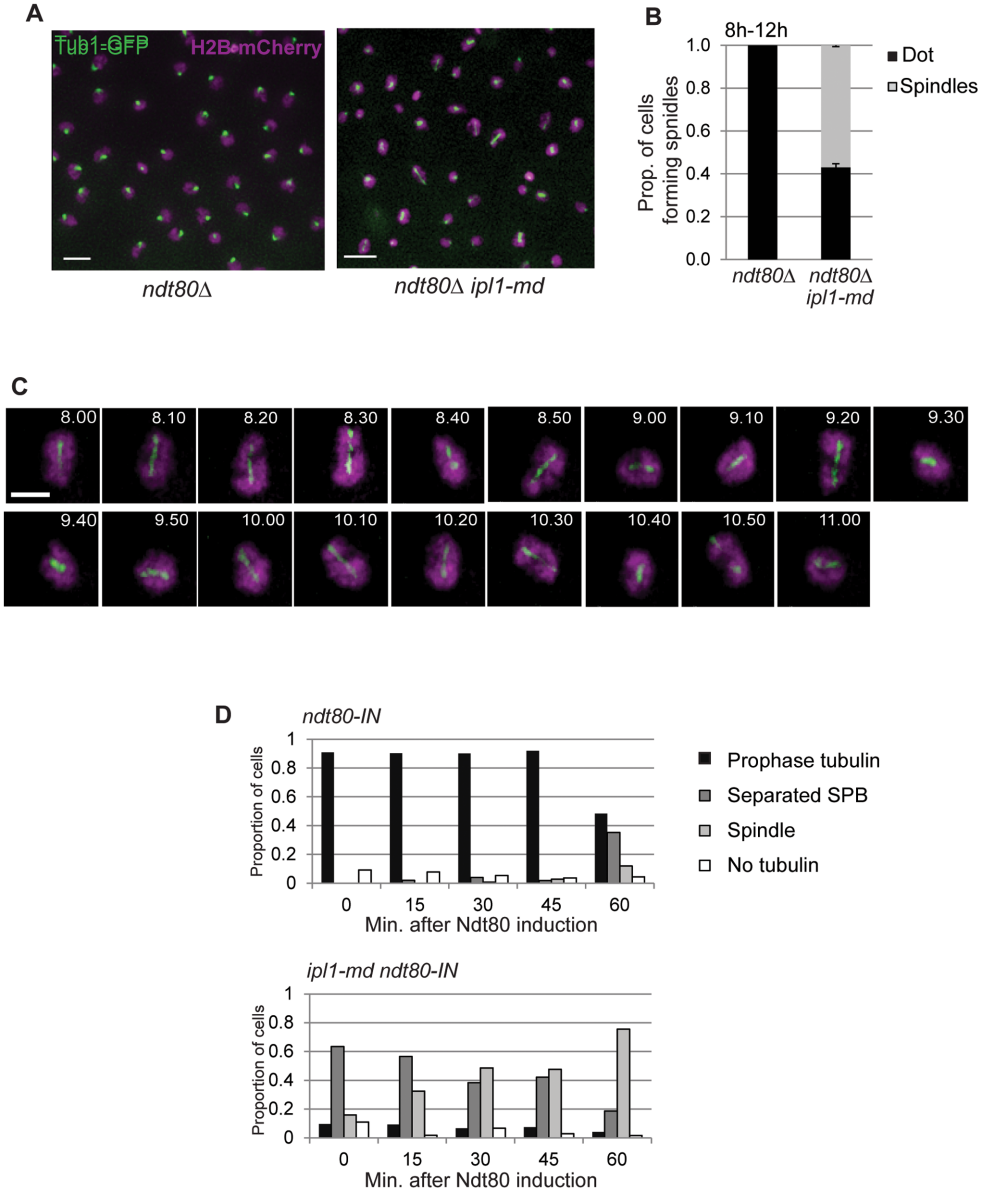
Ipl1 depletion causes precocious formation of spindles in prophase I-arrested *ndt80* mutants. (A) Representative examples of SPB and spindle configurations in *ndt80*Δ and *ndt80*Δ *ipl1-md* mutants. (B) The proportion of cells that formed spindles during the four hours of time-lapse imaging. A small number multipolar spindles were observed; these were added to the ‘spindle’ category. (C) Representative example dynamic behaviour of tubulin during time-lapse imaging of the *ndt80*Δ *ipl1-md* mutant. (D) Spindle formation in *ipl1-md* cells arrested in prophase I (t = 0; 6 hours in sporulation medium), and after release using the *ndt80-IN* system (WT: Y967 and *ipl1-mn* :Y1169). The spindle and SPB conformation were assessed in >100 cells every 15 min. after release from *NDT80* arrest.

**Figure 4:**
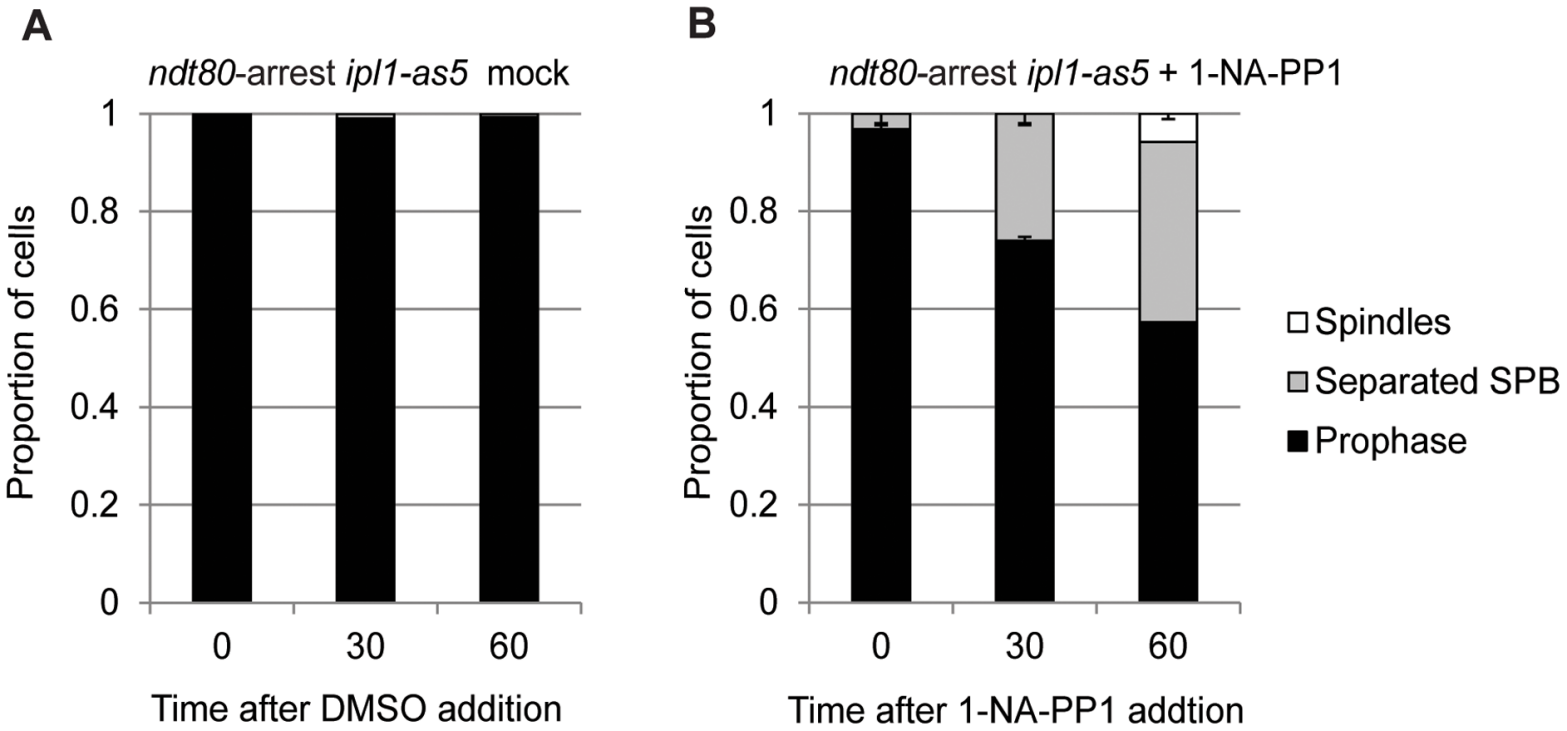
Spindle formation in enhanced when *ipl1-as5* is inhibited during meiotic prophase I arrest (*ndt80*). (A, B) Proportion of *ndt80-*arrested cells carrying the ATP-analogue sensitive *ipl1-as5* allele with separated SPBs or spindles after mock-treatment with DMSO (A) or 50 µM 1-NA-PP1 (B).

If Ipl1 suppresses the formation of spindles during meiotic prophase I, then one would expect *ipl1-md* mutants to form spindles when cells are arrested in prophase I (*ndt80*, Fig. 3). To determine whether this was the case, we followed spindle dynamics (Tub1-GFP) and nuclear separation (H2B-mCherry) during time lapse studies. In agreement with previous observations [23], we observed spindle formation in *ndt80*Δ cells, when Ipl1 was depleted (Fig. 3A,B, Movie S3–S4) or when its kinase activity was inhibited using the *ipl1-as5* allele [31] that renders the kinase sensitive to the ATP analogue, 1-NA-PP1 (Fig. 4). Intriguingly, these spindles appeared to be highly dynamic (Fig. 3C, Movie S5), undergoing several cycles of elongation-collapse. Moreover, spindle elongation and collapse were coordinated with attempts at nuclear separation and relapse (Fig. 3C) suggesting that the spindles are capable of force generation.

We next addressed whether *ipl1-md* mutants are capable of forming spindles when released into M-phase. To do so, we released *ipl1-md* cells from Ndt80-arrest using the *ndt80-IN* (‘*IN*ducible’) allele. In this system, transcription of Ndt80 has been placed under the regulation of the *P_GAL1/10_* promoter. Addition of β-estradiol causes the translocation of Gal4-estrogen receptor fusion protein to the nucleus and induces transcription of genes under the regulation of the *P_GAL1/10_* promoter, including *P_GAL1/10_-NDT80* [13]. In this set up, Clb1 and Clb3 are induced with normal levels and kinetics in the *ipl1-md* mutant relative to wild type [27].

Release from Ndt80 arrest revealed that proficient spindle formation occurred ∼ 15 min after release in the *ipl1-md NDT80-IN* cells, whereas control *NDT80-IN* cells took ∼ 1 hour to display spindles, the time at which Clb1 becomes visible on Western blots [13], [27] (Fig. 3D). Moreover, by 60 min. after Ndt80 induction, when Clb1-CDK is expressed and becomes active [13], [27], nearly 80% of cells had formed spindles in the *ipl1-md NDT80-IN* strain, compared to 10% in the wild type *NDT80-IN* strain (Fig. 3D). Therefore, the efficiency of spindle formation is enhanced after progression into M-phase in the absence of Ipl1. Given the low levels of SPB separation at 30–45 minutes in the *NDT80-IN* wild type control (∼ 5%, Fig. 3D), the enhanced efficiency of spindle formation in the *ipl1-md* strain may be due to the precocious separation of SPBs during the preceding prophase I. This would imply that SPB separation may be a rate-limiting step in spindle formation in meiosis. Alternatively, spindle elongation may be more proficient in *ipl1-md* mutants.

### Ipl1 Suppresses the Formation of Bipolar Spindles in DDR-arrested Cells

**Figure 5:**
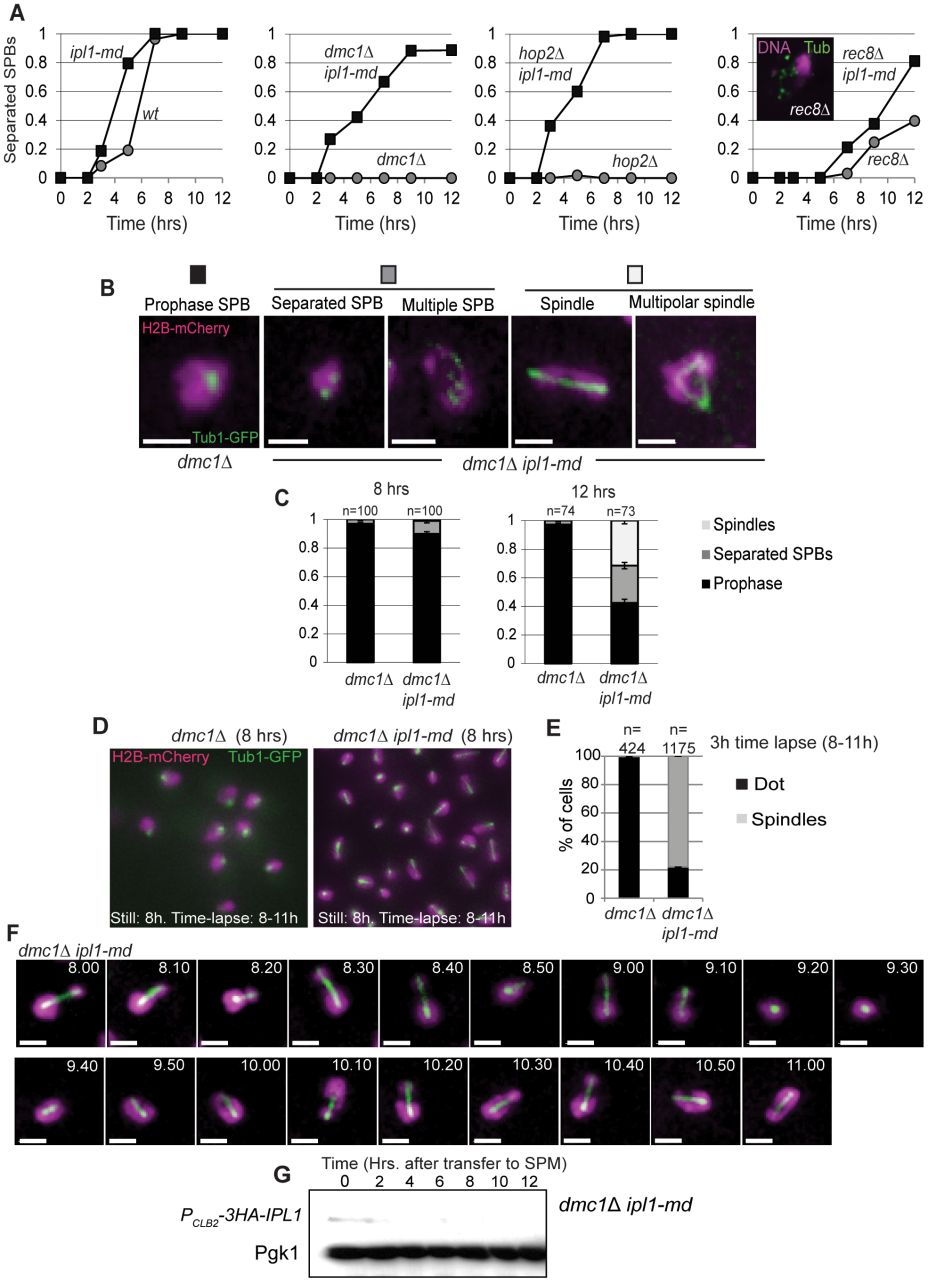
Ipl1 prevents formation of spindles in DDR-arrested cells. (A) Proportion of cells with separated spindle-pole bodies as a function of time. Strains: Wild type (Y940), *ipl1-md* (Y1206), *dmc1*Δ (Y2266), *ipl1-md dmc1*Δ (Y2268), *hop2*Δ (Y2489), *hop2*Δ *ip1-mn* (Y2491) *rec8*Δ (Y2404), *rec8*Δ *ipl1-md* (Y2457). Three independent diploids were assessed, a representative time course is shown for each strain. (B, C) Tubulin configurations observed in *dmc1*Δ *ipl1-md* mutants and their prevalence (C). (D) Representative examples of spindle configurations from a single frame (maximum intensity projection) from time lapse imaging in *dmc1*Δ and *dmc1*Δ *ipl1-md* mutants. (E) The cumulative proportion of cells that formed spindles during the three hours of time-lapse imaging (8–11 h). (F) Representative example dynamic behaviour of tubulin (Tub1-GFP) and DNA (H2B-mCherry) during time-lapse imaging of the *dmc1*Δ *ipl1-md* mutant. (G) Western blot showing that Ipl1 is efficiently depleted in *dmc1*Δ *ipl1-md* cells.

The DDR induces cell cycle arrest and delays the meiotic divisions in response to the accumulation of single-stranded DNA of unrepaired double-strand breaks [15]. We therefore addressed whether Ipl1 is required to prevent spindle formation when cells are arrested by the DDR. To test directly whether Ipl1 inhibits formation of spindles during prophase I arrest, cells were depleted for Ipl1 in three different mutants (*dmc1*Δ, *rec8*Δ, and *hop2*Δ) where the DDR is robustly induced [32], [33], [34]. Ipl1 depletion caused a significant population of *ipl1-md dmc1*Δ and *ipl1-md hop2*Δ cells to separate their SPBs (>80%, Fig. 5A). Even in the *rec8*Δ mutant, where SPBs reduplicate or fragment after prolonged arrest (Fig. 5A, inset), *ipl1-md* significantly shifted the timing and efficiency of SPB separation. We infer that Ipl1 is important in preventing premature SPB separation under DDR-induced arrest.

To determine whether SPB separation was accompanied by spindle formation despite DDR induction in the *ipl1-md* mutant, we examined spindle structures in fixed and live cells using GFP-tagged Tub1 (Fig. 5B–D). Nearly 60% of the *ipl1-md dmc1*Δ cells contained separated SPBs and a third of these (30% overall) contained spindle structures in fixed cells (Fig. 5B,C). Time-lapse imaging revealed that this proportion is a static assessment, which is an underestimate. During a 3 hour time-lapse imaging period, none of the control *dmc1*Δ cells displayed spindle structures (n = 424, Movie S6), whereas >80% of *ipl1-md dmc1*Δ cells (n = 1175) formed at least one spindle structure (Fig. 5D, E, Movie S7) that appeared to display dynamic phases of elongation-collapse (example shown in Fig. 5F, Movie S8). The elongation of the meiotic spindles in *ipl1-md dmc1*Δ cells occurred in concert with attempts at nuclear separation (Fig. 5F).

The spindle dynamics in the *ipl1-md dmc1*Δ cells (Fig. 5F) were reminiscent of that observed in the *ipl1-md ndt80*-arrested cells (Fig. 3C). If these spindles are formed during prophase I, their instability may be due to the lack of anaphase-dependent stabilizing factors [35], inefficient interactions between kinetochores and microtubules [18], or the presence of unresolved joint molecules that prevent chromosome segregation and may cause spindle collapse. Collectively, our data demonstrate that Ipl1 suppresses precocious SPB separation and spindle formation during prophase I, both when cells are repair-proficient (*ndt80*) and when the DDR is induced (*dmc1*, *rec8*, or *hop2*).

### Ipl1 Depletion does not Display Classical Transcriptional or Cell Cycle Bypass of the DDR

At least two explanations could account for the observations that Ipl1 depletion causes the formation of spindles in DDR-arrested recombination mutants (Fig. 5A). *ipl1-md* cells could bypass or fail to initiate the DDR, which would imply a role for Ipl1 in the DDR. Alternatively, Ipl1 may prevent the precocious spindle formation in DDR-arrested cells.

**Figure 6:**
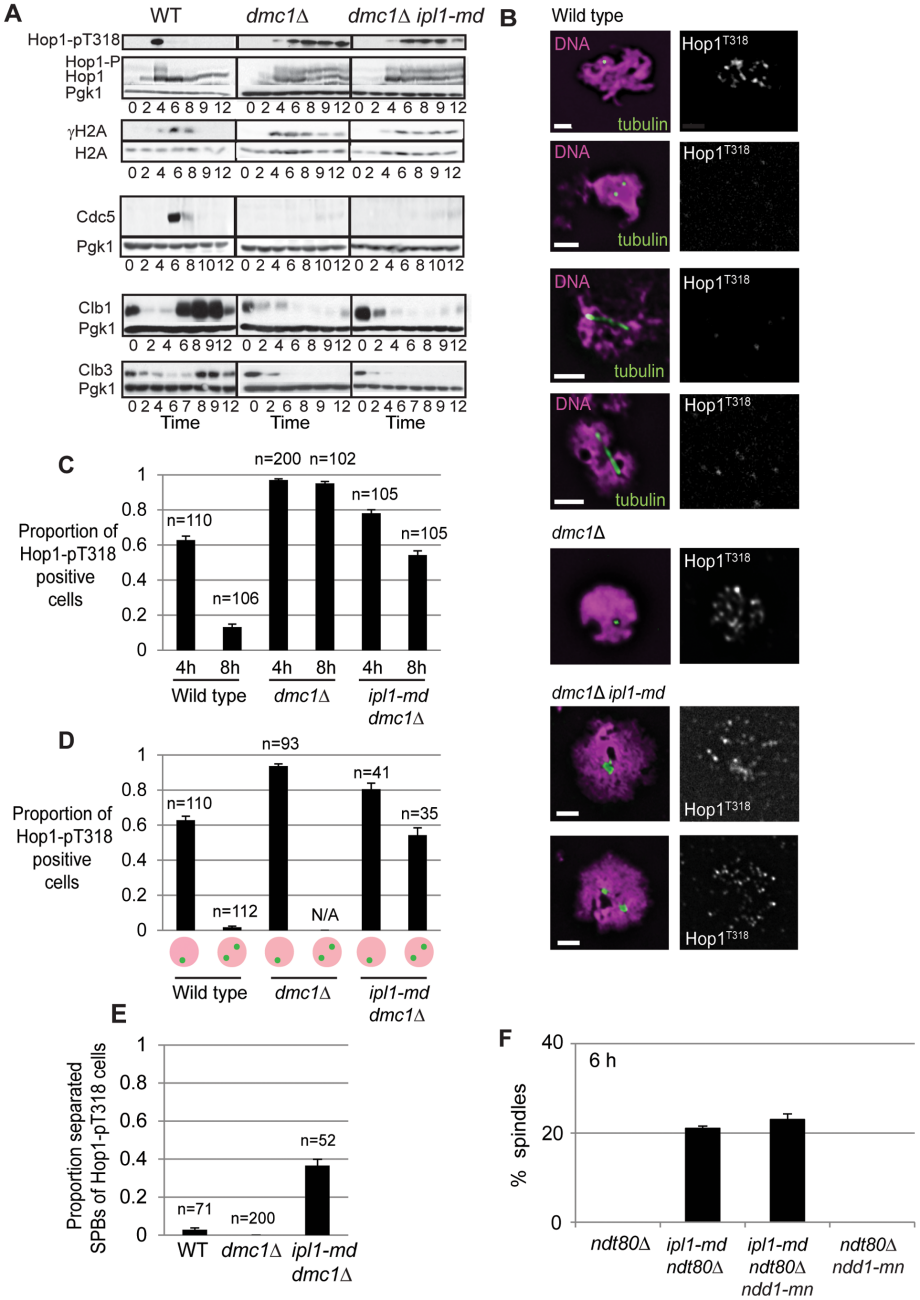
Ipl1 mutants do not bypass the DDR at early time points or display defective regulation of Ndd1. (A) Western blot analysis of Hop1 and γH2A phosphorylation and expression of Cdc5 (MI), Clb1 (MI) and Clb3 (MII) under the regulation of Ndt80. Pgk1 is used as a loading control. Strains: Y4489–Y4494. (B) Examples of phosphorylated Hop1 localization to meiotic chromosomes in wild type, *dmc1*Δ, and *dmc1*Δ *ipl1-md* nuclei. (C) Proportion of nuclei with phospho-Hop1 (T318) staining at 4 h and 8 h. (D) Proportion of nuclei with phospho-Hop1 (T318) staining amongst nuclei with un-separated versus separated SPBs. (E) Proportion of phospho-Hop1 positive nuclei with separated SPBs. (F) Examples of spindle formation in *ipl1-md ndt80*Δ mutant and the % of cells that display spindles in *ndt80*Δ (Y2241), *ipl1-md ndt80*Δ (Y2575), *ipl1-md ndt80*Δ *ndd1-mn* (Y4499), and *ndt80*Δ *ndd1-mn* (Y2646) at 8 hours.

To determine whether *ipl1-md* mutant cells were defective in the activation and maintenance of the DDR, we assessed γH2A and Hop1 phosphorylation, which are regulated by Mec1/ATR and the 9-1-1 clamp [14], [36]. During a meiotic time course, both γH2A and Hop1 phosphorylation appeared and disappeared in wild type cells. In contrast, both γH2A and Hop1 phosphorylation remained high in the *dmc1*Δ mutant as well as in the *ipl1-md dmc1*Δ cells (Fig. 6A). These observations demonstrate that the DDR is activated in the *ipl1-md dmc1*Δ strain, from which we infer that Ipl1 is not required for the initiation of the DDR.

To assess whether the DDR was maintained similarly in the *ipl1-md dmc1*Δ and the *dmc1*Δ strains, we assessed the expression of Cdc5 polo kinase and the M-CDK cyclins, Clb1 and Clb3, which are meiosis I and II-specific, respectively (Fig. 6A, B) [10], [13]. These key cell cycle genes are under the regulation of Ndt80. In both the *dmc1*Δ and *ipl1-md dmc1*Δ cells, only very low levels of Cdc5 and Clb1 appeared at late time points (10–12 hours) compared to wild type. The lack of strong induction of Cdc5 and the Clb1 is not consistent with a classical bypass of DDR maintenance, where the Ndt80-regulon and other M-phase proteins get expressed at high, wild-type levels at early time points [16], [37]. Consistent with this, depletion of the mitotic M-phase transcription factor, Ndd1, did not affect spindle formation in the *ipl1-mn ndt80*Δ strain (Fig. 6F). This rules out that a switch from Ndt80-driven to Ndd1-promoted M-phase transcription occurs in Ipl1-depleted cells.

### Ipl1 Prevents Formation of Spindles in Nuclei with Hop1 Phosphorylation

If Ipl1 suppresses spindle formation in DDR-activated cells, then one should observe spindles or separated SPBs in cells where the DDR is activated. This would predict the existence of meiotic nuclei stained positively for phosphorylated Hop1 and that also contain separated SPBs or spindles. To test whether this was the case, we spread meiotic nuclei and stained with a phospho-specific antibody against Hop1 [38] as well as tubulin (Fig. 6B) in order to determine DDR checkpoint activity on a single-cell basis. In the wild type, 63% (n = 110) of cells were positive for phospho-Hop1 at 4 hours and this decreased to 13% (n = 106) by 8 hours (Fig. 6C), consistent with the progression of cells in meiosis I (100% of cells had separated their SPBs). 63% (n = 110) of cells with a single SPB focus stained positive for phospho-Hop1, whereas only 2% (n = 112) of the cells with separated SPBs were positive for Hop1 phosphorylation (Fig. 6D), demonstrating that Hop1 phosphorylation normally disappears by the time of Ndt80-driven exit from meiotic prophase I. This is consistent with the DDR becoming inactivated prior to transition into M-phase. Conversely, of 71 phospho-Hop1 positive cells, 99% contained un-separated SPBs and only 1% displayed separated SPBs. These observations support the conclusion that progression into M-phase (separation of SPBs) normally occurs concomitantly with the inactivation of the DDR.

In contrast, in the *dmc1*Δ mutant, 97% (n = 200) and 95% (n = 102) of nuclei were positive for Hop1 phosphorylation at 4 and 8 hours, respectively (Fig. 6C). This is consistent with persistent DDR signalling due to the accumulation of extensive single-stranded DNA. All of these nuclei contained un-separated SPBs (Fig. 6E).

In the *ipl1-md dmc1*Δ mutant, despite the slight decrease in phospho-Hop1 positive cells from 4 hours (78%) to 8 hours (54%; Fig. 6C), more than half of the cells (54%, n = 35) with separated SPBs were positive for phospho-Hop1 (Fig. 6D). Moreover, more than a third of nuclei selected for phospho-Hop1 staining (37%, n = 52, Fig. 6E) contained separated SPBs. This demonstrates that spindles can form despite DDR activation when Ipl1 is depleted. Collectively, our data support the conclusion that Ipl1 suppresses the formation of spindles during meiotic prophase I and when the meiotic DDR is active.

### S-CDK is Required and Sufficient to Drive Spindle Formation in the ipl1-md Mutant

**Figure 7:**
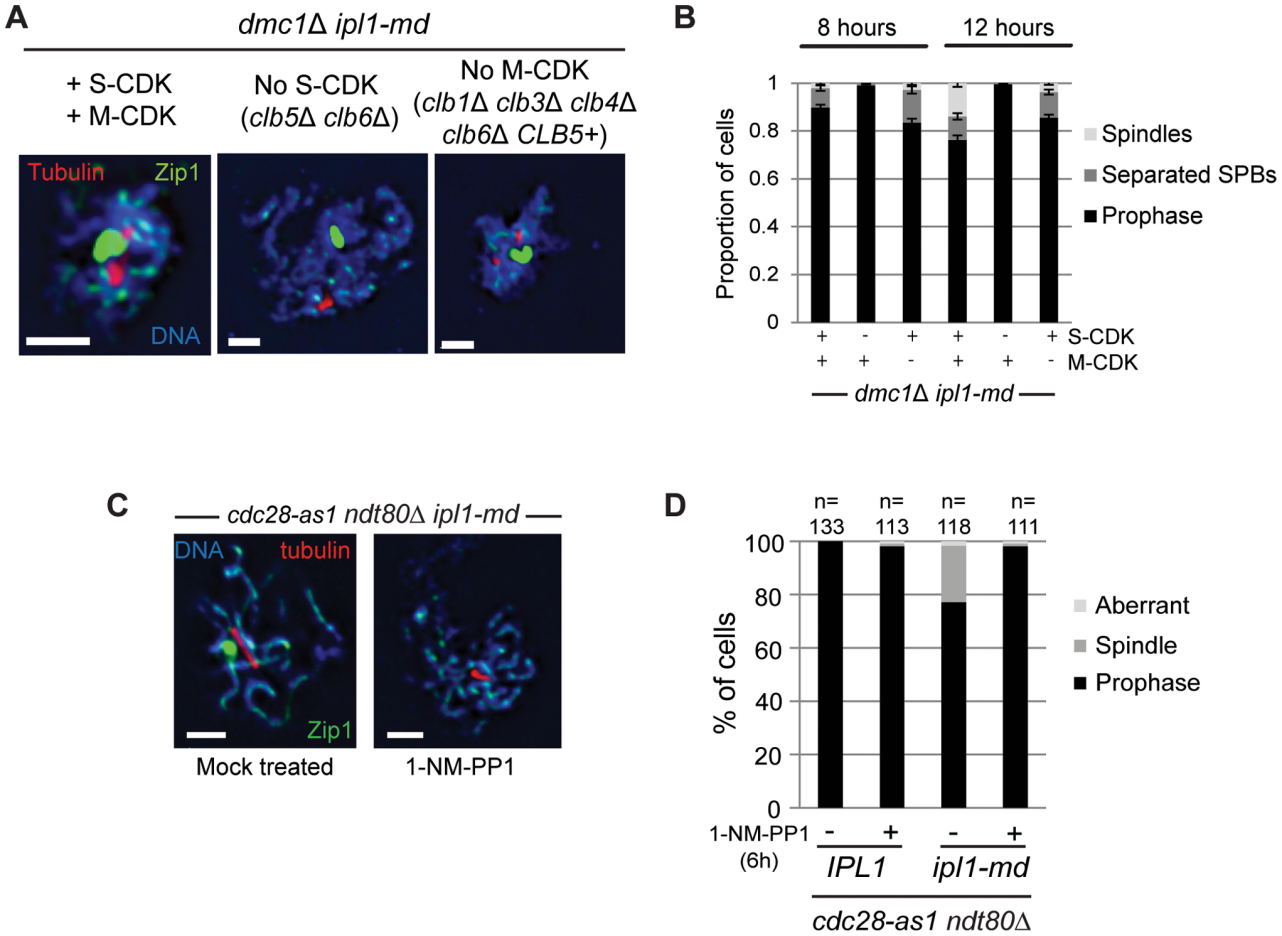
S-CDK is required and sufficient to drive SPB separation and spindle formation during prophase I in *ipl1-md* cells. (A)Images for tubulin and Zip1 staining in *dmc1*Δ *ipl1-md* strains with normal S-CDK and M-CDK (left image), lacking S-CDK activity (*clb5*Δ *clb6*Δ; middle image), or without M-CDK proficient for Clb5 only (*clb1*Δ, *clb3*Δ, *clb4*Δ, *clb6*Δ *CLB5^+^*; right panel). Strains: Y4495, Y4435, and Y4496, respectively. Bars, 2 µm. (B) Quantification on the proportion of fixed cells with spindles and separated SPBs at 8 hours and 12 hours. (C, D) *ipl1-md ndt80*Δ *cdc28-as1* (Y2577) cells were treated with either 50 µM 1-NM-PP1 (+) or solvent only (DMSO) (−) to inhibit Cdc28/CDK kinase activity at 6 hours, when spindles have formed in at least 20% of *ipl1-md ndt80*Δ cells. Examples of spread, meiotic nuclei are shown to the left. Note that there was no effect on inhibiting Cdc28-as1 in *ndt80*Δ alone bars, 2 µm. The graph shows that Quantification of prophase spreads with spindles or aberrant spindle pole structures (*e.g.* multipolar spindles).

The hypothesis that Ipl1 suppresses spindle formation during meiotic prophase I when the meiotic DDR is intact makes three clear predictions. First, if spindle formation occurs in cells that are biochemically in meiotic prophase I, then S-CDK would be expected to drive the formation of the spindles, since M-CDK is presumably inactive. This predicts that deleting S-CDK activity (*clb5*Δ *clb6*Δ) should abrogate spindle formation in *ipl1-md dmc1*Δ cells. To test this prediction, we generated an *ipl1-md dmc1*Δ *clb5*Δ *clb6*Δ quadruple mutant and assessed spindle formation (Fig. 7A,B). Without S-CDK activity, none of the cells displayed spindles and only a very minor fraction (<1%) showed a doublet of SPBs (e.g. middle image in Fig. 7A). This strongly suggests that the spindle formation in the *ipl1-md* mutant is dependent upon S-CDK activity, when the meiotic DDR is active.

Second, if S-CDK drives spindle formation, then S-CDK activity should be sufficient to cause spindle formation in the *ipl1-md dmc1*Δ mutant. To test whether this was the case, we assessed spindle formation in this mutant when the M-CDKs were deleted (*clb1*Δ *clb3*Δ *clb4*Δ *clb6*Δ). In this strain where Clb5-CDK drives meiosis and M-CDK is absent (*ipl1-md dmc1*Δ *clb1*Δ *clb3*Δ *clb4*Δ *clb6*Δ *CLB5^+^*), spindle formation occurred with similar efficiency compared to the *ipl1-md dmc1*Δ strain that contained intact M-CDK (Fig. 7A, B). These observations support the notion that Clb5-CDK is sufficient to drive spindle formation, when Ipl1 is depleted.

Finally, if S-CDK promotes the formation of spindles in normal, DNA repair proficient cells (*DMC1*), then inhibiting CDK activity should abolish spindle formation during meiotic prophase (*ndt80*) in Ipl1-depleted cells. To test whether the spindle formation depended upon CDK activity, we inhibited the single cell cycle CDK in budding yeast (Cdc28) in prophase I arrested cells (*ndt80*). To this end, we used the bio-orthogonal approach of modifying the ATP binding site of Cdc28 (*cdc28-as1*) and challenging cells with a modified ATP analogue (1-NM-PP1) that specifically inhibits Cdc28-as1, but not other ATPases [39]. In the mock-treated *ipl1-md ndt80*Δ *cdc28-as1* strain, we observed 21% (±3.7%) of cells with spindles at 8 hours (fixed cells; Fig. 7C, D). In contrast, when cells were treated with the ATP analogue to inhibit Cdc28/CDK activity, the percentage of cells with spindles was reduced to 3% (±2.1%; Fig. 7C,D). This is consistent with CDK activity being critical for spindle formation during meiotic prophase I. Moreover, since the inhibitor was added after spindle formation had initiated in the *ipl1-md ndt80*Δ cells, continuous CDK activity appears to be important for spindle formation. One possibility is that CDK activity is required continuously due to the cycles of elongation-collapse that the *ipl1-md* spindles undergo (Fig. 3C, 5F).

Collectively, our data show that S-CDK is sufficient and necessary to drive spindle formation during prophase I arrest in budding yeast meiosis, when Ipl1 is depleted. From this we infer that Ipl1 is required to suppress S-CDK-mediated spindle formation during meiotic prophase I in arrested cells (*ndt80*Δ) and during DDR-mediated arrest, when double-strand break repair is defective (*dmc1*Δ).

### Efficiency of Spindle Formation in Ipl1-depleted Cells is Enhanced by Cdc5 Polo Kinase

**Figure 8:**
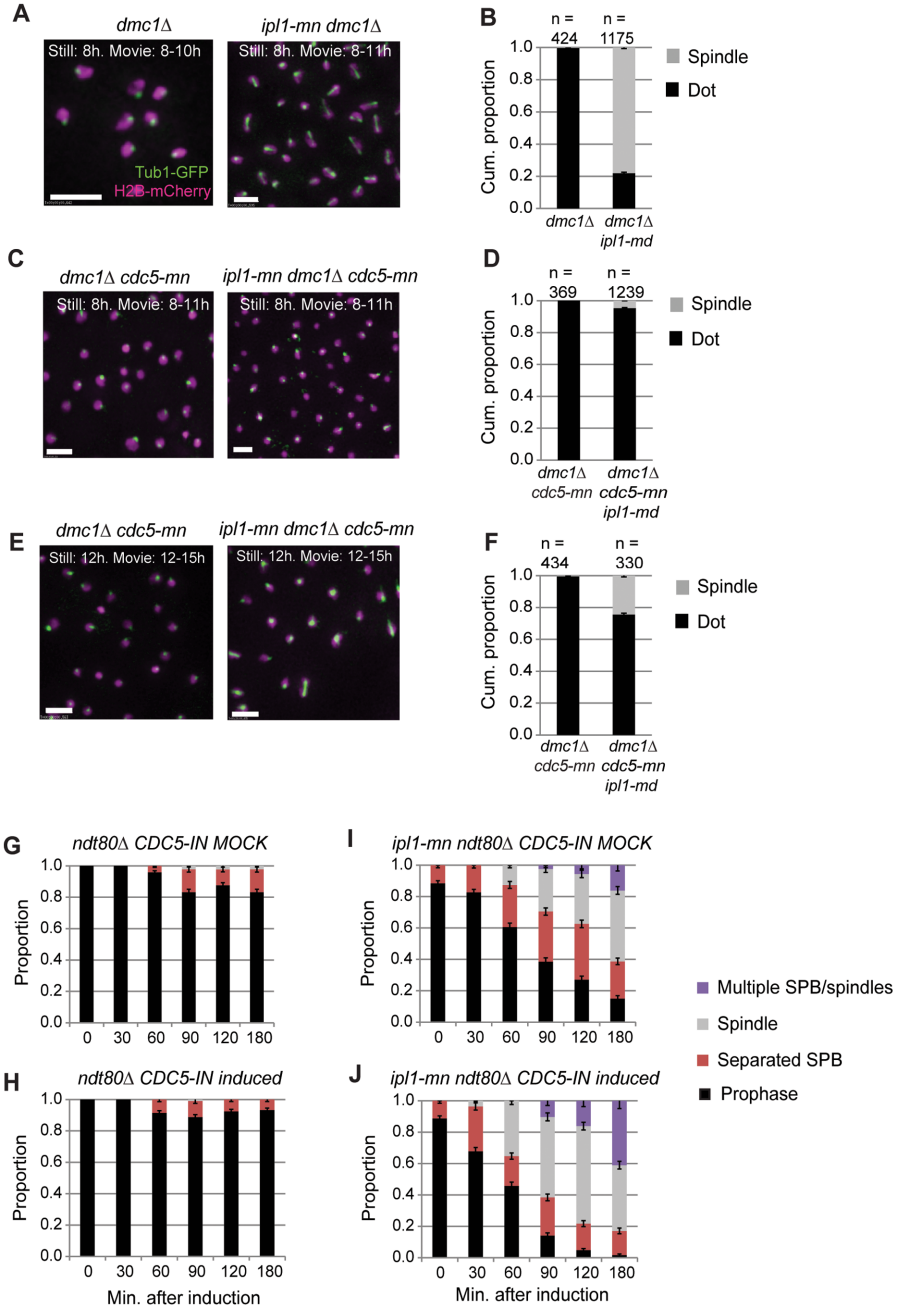
Meiotic depletion of Cdc5 causes delayed spindle formation in *ipl1-md* cells. (A,B) Examples of spindle formation (Tub1-GFP) and nuclear dynamics (H2B-mCherry) in *dmc1*Δ (Y4301), *ipl1-mn dmc1*Δ (Y4304). Bar: 5 µm. The cumulative proportion of cells forming spindle structures during the time lapse are shown in the graph to the right (B). (C,D) Examples of spindle formation (Tub1-GFP) and nuclear dynamics (H2B-mCherry) *dmc1*Δ *cdc5-mn* (Y4405; Movie S6), and *ipl1-mn dmc1*Δ *cdc5-mn* (Y4398; Movie S9–10). The cumulative proportion of cells forming spindle structures during the time lapse from 8–11 hours are shown in the graph (D). (E,F) Examples of spindle formation (Tub1-GFP) and nuclear dynamics (H2B-mCherry) *dmc1*Δ *cdc5-mn* (Y4405; Movie S11), and *ipl1-mn dmc1*Δ *cdc5-mn* (Y4398; Movie S11–12). The cumulative proportion of cells forming spindle structures during the time lapse from 12–15 hours is shown in the graph (F). (G, H) Population dynamics of SPB separation and spindle formation in prophase I arrested cells (*ndt80*), where mock-treatment (K) or induction of *CDC5* (L) occurred. *CDC5*-*IN* (*P_GAL1/10_-CDC5 GAL4.ER* has been described previously (Souranajan and Lichten, 2008; Jordan et al. 2009) and strains also carried a wild-type copy of *CDC5*. (I, J) Population dynamics of SPB separation and spindle formation in prophase I arrested cells with Ipl1 depleted (*ipl1-md ndt80*), where mock-treatment (D) or induction of *CDC5* (E) occurred, as in (K,L).

Cdc5 polo kinase is important for the timely separation of SPBs in both mitosis and meiosis of budding yeast [21], [40]. In meiotic prophase I, Cdc5 levels are kept low due to degradation by the APC^Ama1^ [16], until Ndt80 induction, upon which Cdc5 levels accumulate (Fig. 6A) [10]. Depletion of Cdc5 during prophase I leads to defects in Ndt80 production [41]. To understand the requirement for polo kinase in meiotic spindle formation when Ipl1 is depleted, we assessed SPB dynamics in *ipl1-md* mutants that also lacked polo kinase activity (*cdc5-meiotic depletion*). In the *ipl1-md dmc1*Δ mutant, the cumulative proportion of cells that formed a spindle during 3 hours of time-lapse imaging was ∼ 80% (Fig. 8B, Movie S7). In contrast, when Cdc5 was depleted in this background, SPB separation and spindle appearance was significantly reduced (5% of cells; Fig. 8C,D, Movie S9–S10). Only from 12 hours onwards, after a 4 hour delay, did a significant proportion of *ipl1-md dmc1*Δ *cdc5-mn* cells form spindles (Fig. 8E,F, Movie S11–S12). This delay is similar to that reported in ensemble population studies of *cdc5* alone [21]. Unlike the prophase I spindles formed in the *ipl1-md dmc1*Δ of *ipl1-md nt80* mutants, these spindles were not dynamic, but appeared to elongate before disassembling with separated DNA masses (Movie S13). From these observations, we infer that although even low levels of Cdc5 may be sufficient to promote SPB separation, when Ipl1 activity is low or suppressed.

If Cdc5 promotes the efficiency of spindle formation during meiotic prophase I in *ipl1-md* cells, then ectopic expression of Cdc5 in *ndt80*-arrested prophase cells should enhance spindle formation in *ipl1-md* mutant. Ectopic overexpression of Cdc5 on its own is insufficient to drive spindle formation in *ndt80* arrested cells (Sourirajan and Lichten 2008, and Fig. 8H). However, when Cdc5 was induced in the Ipl1-depleted cells (*ndt80*Δ *ipl1-md CDC5-IN*), enhanced efficiency of SPB separation and spindle formation was observed compared to mock induction (Fig. 8J versus I, respectively; P<0.01, G-test). These experiments demonstrates that Cdc5 contributes towards the efficient formation of spindles when Ipl1 is depleted. Furthermore, they show that, at least in part, induction of Cdc5 has no effect due to the presence of Ipl1.

**Figure 9:**
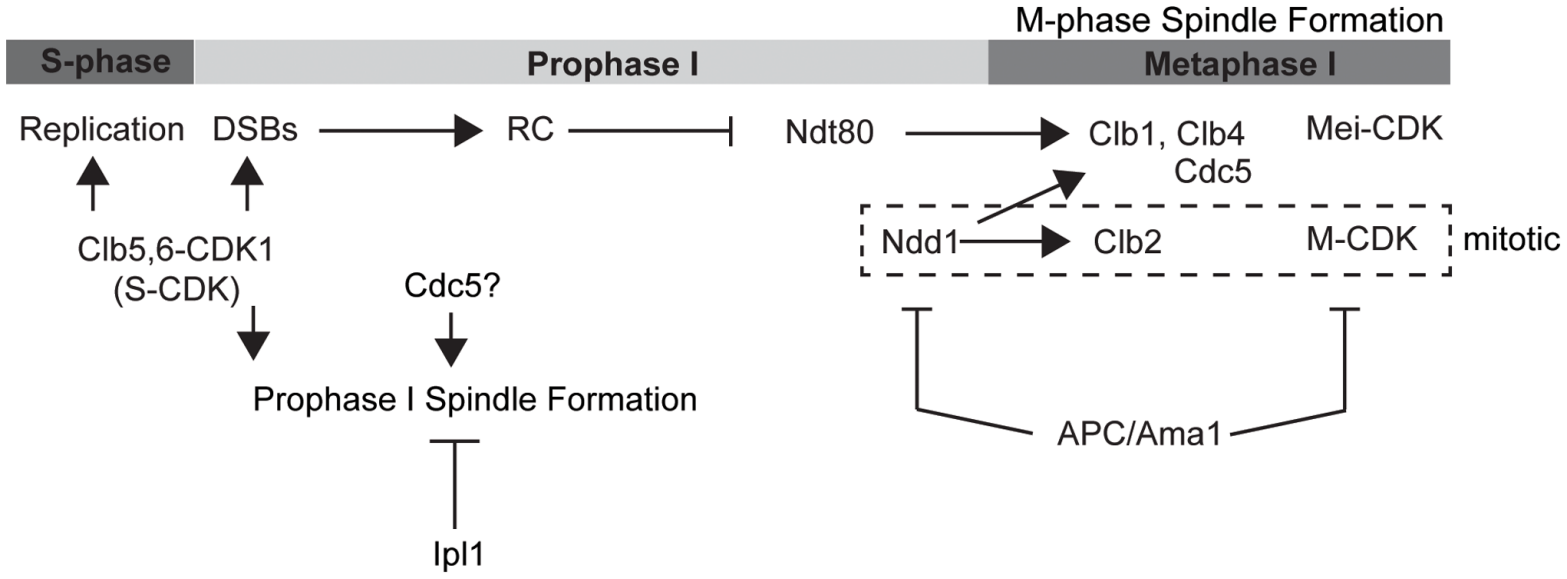
Model of entry into meiosis I, which is regulated by M-CDK (Clb1, Clb3, and Clb4). S-CDK (Clb5, Clb6) is required for induction of meiotic recombination.

Coordination of spindle formation and chromosome restructuring in preparation for chromosome segregation is essential during meiosis. In this work, we have identified a novel and unexpected role for Ipl1 during meiotic prophase I in suppressing spindle formation in both prophase I-arrested (*ndt80*Δ) and DDR-arrested (*dmc1*Δ) cells. Specifically, Ipl1 activity is required to suppress or counteract spindle formation by S-CDK and when Cdc5 activity is low. Repressing the formation of spindles by S-CDK during meiotic prophase I is essential, because S-CDK is active and indeed required for the initiation of meiotic recombination [12]. Many studies of Aurora kinases to date have revealed critical functions in the formation and stabilization of spindles. Our findings and those of Kim *et al*. [24] reveal another function in the suppression of precocious spindle formation. Ipl1 is also important for the disassembly of the outer kinetochores during early stages of meiotic prophase I, which prevents ends-on chromosomal attachments to microtubules [28]. Thus, Ipl1 has a dual function in suppressing inappropriate attachment of immature meiotic chromosomes to spindles during meiotic prophase I. Our data show that Ipl1 prevents spindle formation facilitated by S-CDK and to lesser extent, Cdc5, during prophase I. The active suppression of S-CDK-mediated and Cdc5 polo kinase-driven spindle formation during meiotic prophase I, or when the DDR is active (illustrated in Figure 9), is consistent with findings that ectopic expression of Cdc5 or Clb5 during prophase I is not sufficient to cause spindle formation [18], [20]. In particular, Clb5 overexpression in prophase I leads to an enhancement of CDK activity that is similar in magnitude to that observed for the meiosis II specific M-phase cyclin, Clb3 [18]. However, unlike Clb3, overexpression of Clb5 does not induce spindle formation [18], presumably due to the presence of Ipl1.

Our findings that CDK and polo kinase can drive or enhance spindle formation in prophase I (when Ipl1 is depleted or inactivated) is analogous to recent reports that CDK- and polo kinase promote centrosome separation during interphase in higher eukaryotes (mitotic cell cycle) [42], [43], [44]. Our data further demonstrate that M-CDK and high protein levels of Cdc5 (both induced by Ndt80 upon entry into M-phase) are not a *de facto* requirement for spindle formation in budding yeast meiosis. Instead, S-CDK and low levels of Cdc5 are sufficient to drive spindle elongation, but only in the absence of Ipl1. In a separate study, Kim *et al*. [24] showed that Ipl1 may prevent precocious spindle formation by blocking Clb4 localisation at spindle pole bodies. This raises the intriguing possibility that Ipl1 functions directly at SPBs in a localized manner to prevent SPB separation and spindle formation by S-CDK (Clb5) during meiotic prophase I. Another possibility is that Ipl1’s role in SPB cohesion in itself [23] prevents Cdc5- and S-CDK-mediated spindle formation. For example, if SPB separation is the rate limiting step during spindle formation in budding yeast, then loss of SPB cohesion might be sufficient to trigger spindle formation by S-CDK and Cdc5.

## Materials and Methods

### Strains and Meiotic Time Course Experiments

All strains were generated in the SK1 background and are shown in Table S1. Diploid strains were generated from freshly mated haploids, individual diploid colonies were then incubated in 5 ml of liquid rich medium and transferred to pre-sporulation medium (SPS). Cells were subsequently resuspended in 2% liquid potassium acetate medium (KAC) to induce meiosis [27]. All experiments were performed at 30°C. We observed day-to-day variation on time courses and therefore carried out all wild-type versus mutant analyses on the same day.

### Time-lapse Imaging, Image Rendering, and Image Analysis

All time-lapse imaging took place in CellAsics Y0D microfluidics chambers, with conditions on a pDV with solid-state illumination and detection by the Cascade 1K EMCCD. All conditions were optimized for Nyquist sampling and illumination times were tested on wild type cells to ensure sporulation. Specific conditions for imaging are being published elsewhere. The movies were all rendered in Softworx. 3D measurements of spindle lengths were carried out in Imaris. All images of the live cells are maximum intensity projections. For meiotic spreads, images were prepared from.dv files in Adobe Photoshop files in Softworx and rendered in Photoshop CS5. Only total brightness/contrast levels were altered (not alpha).

### Protein Extraction, Western Blot Analysis and Antibodies

Protein extraction by TCA and Western blot analysis were carried out as described previously [27]. For Western blot analysis, blots were probed with the appropriate antibodies followed by HRP-conjugated secondary antibodies (DAKO, 1∶2000). HRP activity was detected using Pierce ECL Western Blotting Substrate followed by exposure to Amersham Hyperfilm ^TM^ECL or using the Image Quant™ LAS 4000 imaging system. Antibodies used for Western blot analysis were as follows:

Mouse (monoclonal) anti-HA (12CA5), 1∶1000, S. Ley, NIMR, UK. Rabbit (polyclonal) anti-Hop1, 1∶1500, F. Klein, MFPL, Vienna, Austria. Rabbit (polyclonal) anti-phosphoT318-Hop1, 1∶500, Cambridge Research Biomedicals. Mouse (monoclonal) anti-Myc (9E10), 1∶1000, S. Ley, NIMR, UK. Goat (polyclonal) anti-Cdc5 (YN019), 1∶1000, Santa Cruz Biotech (sc-6732). Rabbit Anti-γH2A (Dr. Jessica Downs, 1∶1000). Rabbit Anti-H2A (Dr. Jessica Downs, 1∶1000). Mouse Anti-Pgk1 (Invitrogen 459250, 1∶20,000). Rat anti-tubulin (YOL034W (1∶400, Novus Biologicals).

Rabbit (polyclonal) anti-Zip1, 1∶100, Hoffmann lab [45], [46]. Antibodies used for immunofluorescence were as follows:

Guinea pig anti-phosphoT298-Hop1, 1∶100, Cambridge Research Biomedicals.

Rabbit (polyclonal) anti-phosphoT318-Hop1, 1∶500, Cambridge Research Biomedicals. Secondary antibodies were used as described previously, all from Jackson Immunoresearch [45], [46].

### Statistics

Box-and-whisker plots were rendered in R (www.r-project.org) and the vertical bar denotes the median value. Error bars around proportions were calculated as √p×[1-p]/n, where n the number of observations.

## Supporting Information

Table S1 Strain list.
(PDF)

Movie S1 **SC disassembly in wild type, matching the stills in** **Fig. 1B** **.**
(MOV)

Movie S2 **SC disassembly in** ***ipl1-mn*** **, matching the stills in** **Fig. 1B** **.**
(MOV)

Movie S3 **Tub1-GFP and H2B-mCherry dynamics in** ***ndt80*** **Δ, matching stills in** **Fig. 1B** **.**
(MOV)

Movie S4 **Tub1-GFP and H2B-mCherry dynamics in** ***ndt80*** **Δ** ***ipl1-md*** **, matching stills in** **Fig. 1B** **.**
(MOV)

Movie S5 **Tub1-GFP and H2B-mCherry dynamics of a single cell** ***ndt80*** **Δ** ***ipl1-md*** **, matching stills in** **Fig. 1E** **.**
(MOV)

Movie S6 **Tub1-GFP and H2B-mCherry dynamics in** ***dmc1*** **Δ, matching stills in** **Fig. 2D** **.**
(MOV)

Movie S7 **Tub1-GFP and H2B-mCherry dynamics in** ***ipl1-mn dmc1*** **Δ, matching stills in** **Fig. 2D** **.**
(MOV)

Movie S8 **Tub1-GFP and H2B-mCherry dynamics of a** ***ipl1-mn dmc1*** **Δ single cell, matching stills in** **Fig. 2F** **.**
(MOV)

Movie S9 **Tub1-GFP and H2B-mCherry dynamics** ***dmc1*** **Δ** ***cdc5-mn*** **at 8** **h, matching stills in** **Fig. 5B** **.**
(MOV)

Movie S10 **Tub1-GFP and H2B-mCherry dynamics** ***ipl1-mn dmc1*** **Δ** ***cdc5-mn*** **at 8 h, matching stills in** **Fig. 5B** **.**
(MOV)

Movie S11 **Tub1-GFP and H2B-mCherry dynamics** ***dmc1*** **Δ** ***cdc5-mn*** **at 12 h, matching stills in** **Fig. 5C** **.**
(MOV)

Movie S12 **Tub1-GFP and H2B-mCherry dynamics** ***ipl1-mn dmc1*** **Δ** ***cdc5-mn*** **at 12 h, matching stills in** **Fig. 5C** **.**
(MOV)

Movie S13 **Close up of a single cell displaying nuclear separation and multipolar spindles in the** ***ipl1-mn dmc1*** **Δ** ***cdc5-mn*** **at 12 h.**
(MOV)
