# Supplementary material for: Ipl1/Aurora Kinase Suppresses S-CDK-Driven Spindle Formation during Prophase I to Ensure Chromosome Integrity during Meiosis
Source: PLoS One. 2013 Dec 27;8(12):e83982. doi: 10.1371/journal.pone.0083982 (PMC3873974; doi:10.1371/journal.pone.0083982)
Supplement: Table S1 — Strain list. (PDF) [file pone.0083982.s001.pdf]

Diploid no.	Strain no.	Genotype	*MAT*
Y940	Y885	Wild type	a
	Y886	Wild type	α
Y1206	Y1192	*pCLB2-3HA-IPL1 (KANMX6)*	a
	Y1193	*pCLB2-3HA-IPL1 (KANMX6)*	α
Y2241	Y2185	*ndt80::HYG, ER-GAL4::URA3*	a
	Y2186	*ndt80::HYG, ER-GAL4::URA3*	α
Y2266	Y2254	*dmc1::HYG*	a
	Y2255	*dmc1::HYG*	α
Y2404	Y2236	*rec8::KANMX4*	a
	Y2237	*rec8::KANMX4*	α
Y2457	Y2422	*pCLB2-3HA-IPL1 (KANMX6) rec8::KANMX4*	a
	Y2423	*pCLB2-3HA-IPL1 (KANMX6) rec8::KANMX4*	α
Y2489	Y2474	*hop2::KANMX4*	a
	Y2475	*hop2::KANMX4*	α
Y2491	Y2478	*pCLB2-3HA-IPL1 (KANMX6) hop2::KANMX4*	a
	Y2479	*pCLB2-3HA-IPL1 (KANMX6) hop2::KANMX4*	α
Y2575	Y2541	*pCLB2-3HA-IPL1 (KANMX6) ndt80::HYG*	a
	Y2542	*pCLB2-3HA-IPL1 (KANMX6) ndt80::HYG*	α
Y2656	Y2610	*pCLB2-3HA-NDD1* (*KANMX6*), Estrogen Receptor-GAL4TF (*URA3*) , proGAL1-*NDT80* (TRP1)	a
	Y2645	*pCLB2-3HA-NDD1* (*KANMX6*), Estrogen Receptor-GAL4TF (*URA3*) , proGAL1-*NDT80* (TRP1)	α
Y2572	Y2597	*REC8-GFP-URA3, PDS1-tdTomato-KlTRP1, CNM67-3mCherry-NATMX4*	α
	Y2598	*REC8-GFP-URA3, PDS1-tdTomato-KlTRP1, CNM67-3mCherry-NATMX4*	a
Y2577	Y2545	*pCLB2-3HA-IPL1 (KANMX6), cdc28-as1, ndt80::HYG*	a
	Y2546	*pCLB2-3HA-IPL1 (KANMX6), cdc28-as1, ndt80::HYG*	α
Y2852	Y2814	*SMC5-13MYC (KANMX6), pCLB2-3HA-TOP1 (KANMX6)*	a
	Y2815	*SMC5-13MYC (KANMX6), pCLB2-3HA-TOP1 (KANMX6)*	α
Y3606	Y3604	*Htb1-mcherry-NATMX4, PDS1-tdTomato-KlTRP1, his3::HIS3p-GFP-TUB1-HIS3*	a
	Y3605	*Htb1-mcherry-NATMX4, PDS1-tdTomato-KlTRP1, his3::HIS3p-GFP-TUB1-HIS3*	α
Y4044	Y4042	*ZIP1:700:GFP, PDS1-tdTomato-KlTRP1, CNM67-3mCherry-NATMX4*	a
	Y4043	*ZIP1:700:GFP, PDS1-tdTomato-KlTRP1, CNM67-3mCherry-NATMX4*	α
Y4047	Y4045	*ZIP1:700:GFP, PDS1-tdTomato-KlTRP1, CNM67-3mCherry-NATMX4, pCLB2-3HA-IPL1 (KANMX6)*	a
	Y4046	*ZIP1:700:GFP, PDS1-tdTomato-KlTRP1, CNM67-3mCherry-NATMX4, pCLB2-3HA-IPL1 (KANMX6)*	α
Y4301	Y4299	*DMC1::HYG, Htb1-mcherry-NATMX4, PDS1-tdTomato-KlTRP1, his3::HIS3p-GFP-TUB1-HIS3*	a
	Y4300	*DMC1::HYG, Htb1-mcherry-NATMX4, PDS1-tdTomato-KlTRP1, his3::HIS3p-GFP-TUB1-HIS3*	α

Diploid no.	Strain no.	Genotype	*MAT*
Y4304	Y4302	*pCLB2-3HA-IPL1 (KANMX6), DMC1::HYG, Htb1-mcherry-NATMX4, PDS1-tdTomato-KlTRP1, his3::HIS3p-GFP-TUB1-HIS3*	a
	Y4303	*pCLB2-3HA-IPL1 (KANMX6), DMC1::HYG, Htb1-mcherry-NATMX4, PDS1-tdTomato-KlTRP1, his3::HIS3p-GFP-TUB1-HIS3*	α
Y4398	Y4396	*pCLB2-3HA-CDC5 (KANMX6), pCLB2-3HA-IPL1 (KANMX6), dmc1::HYG, Htb1-mcherry-NATMX4, his3::HIS3p-GFP-TUB1-HIS3*	a
	Y4397	*pCLB2-3HA-CDC5 (KANMX6), pCLB2-3HA-IPL1 (KANMX6), dmc1::HYG, Htb1-mcherry-NATMX4, his3::HIS3p-GFP-TUB1-HIS3*	α
Y4406	Y4404	*pCLB2-CDC5::kanMX6, dmc1::HYG, Htb1-mcherry-NATMX4, PDS1-tdTomato-KlTRP1, his3::HIS3p-GFP-TUB1-HIS3*	a
	Y4405	*pCLB2-CDC5::kanMX6, dmc1::HYG, Htb1-mcherry-NATMX4, PDS1-tdTomato-KlTRP1, his3::HIS3p-GFP-TUB1-HIS3*	α
Y4489	Y2308	*CLB1-9MYC (TRP1)*	a
	Y2309	*CLB1-9MYC (TRP1)*	α
Y4490	Y1389	*CLB3-13MYC (HIS3MX6)*	a
	Y1423	*CLB3-13MYC (HIS3MX6)*	α
Y4491	Y2555	*dmc1::HYG (HPH), CLB1-9MYC (TRP1)*	a
	Y2556	*dmc1::HYG (HPH), CLB1-9MYC (TRP1)*	α
Y4492	Y2559	*dmc1::HYG (HPH), CLB3-13MYC (HIS3MX6)*	a
	Y2560	*dmc1::HYG (HPH), CLB3-13MYC (HIS3MX6)*	α
Y4493	Y2557	*pCLB2-3HA-IPL1 (KANMX6), dmc1::HYG (HPH), CLB1-9MYC (TRP1)*	a
	Y2558	*pCLB2-3HA-IPL1 (KANMX6), dmc1::HYG (HPH), CLB1-9MYC (TRP1)*	α
Y4494	Y2561	*pCLB2-3HA-IPL1 (KANMX6), dmc1::HYG (HPH), CLB3-13MYC (HIS3MX6)*	a
	Y2562	*pCLB2-3HA-IPL1 (KANMX6), dmc1::HYG (HPH), CLB3-13MYC (HIS3MX6)*	α
Y4495	Y2258	*pCLB2-3HA-IPL1 (KANMX6), dmc1::HYG (HPH)*	a
	Y2259	*pCLB2-3HA-IPL1 (KANMX6), dmc1::HYG (HPH)*	α
Y4496	Y4452	*clb1-clb6::KANmx6, clb3::natmx4, clb4::HPHmx4, pCLB2-3HA-IPL1 (KANMX6), DMC1::HYG*	a
	Y4454	*clb1-clb6::KANmx6, clb3::natmx4, clb4::HPHmx4, pCLB2-3HA-IPL1 (KANMX6), DMC1::HYG*	α
Y4499	Y4497	*pCLB2-3HA-IPL1 (KANMX6), ndt80::HYG, pCLB2-3HA-NDD1 (KANMX6)*	a
	Y4498	*pCLB2-3HA-IPL1 (KANMX6), ndt80::HYG, pCLB2-3HA-NDD1 (KANMX6)*	α

all strains are SK1 isogenic for *his3::hisG, leu2::hisG, trp1::hisG, lys2, ura3, ho::LYS2*
